# Donor Characteristics in Graft Detachment after Posterior Lamellar Keratoplasty for Fuchs Endothelial Dystrophy and Bullous Keratopathy

**DOI:** 10.3390/jcm13061593

**Published:** 2024-03-11

**Authors:** Nicola Cardascia, Flavio Cassano, Valentina Pastore, Maria Gabriella La Tegola, Alessandra Sborgia, Francesco Boscia, Giovanni Alessio

**Affiliations:** 1 UOC Oftalmologia Universitaria, Ospedale Universitario Consorziale Policlinico Giovanni XXIII di Bari, Università degli Studi di Bari “Aldo Moro”, 70121 Bari, Italy; cardascia@hotmail.com (N.C.); valentinapastore@hotmail.it (V.P.); mariagabriella.lategola@uniba.it (M.G.L.T.);; 2 UOC Oftalmologia, Ospedale Vito Fazzi, 73100 Lecce, Italy

**Keywords:** corneal dystrophies, corneal transplantation, diseases of the ocular surface, phacoemulsification, post-operative anterior segment problems, complications of refractive surgery

## Abstract

**Background:** Descemet Membrane Endothelial Keratoplasty (DMEK) has been widely adopted to treat Fuchs endothelial dystrophy (FED) and Bullous keratopathy (BK). Graft detachment (GD) is one of the common earliest post-operatory complications, and it is usually recovered by Air Rebubbling (ARB). **Methods:** Retrospectively, we investigated predictive factors related to GD between January 2016 and March 2020, a pre-COVID era, in 72 patients, 72 eyes, and their donors’ lamellar characteristics, focusing on donor’s cause of death. The patients were divided according to the posterior lamellar keratoplasty technique adopted. **Results:** GD and consequent ARB were most common but not significantly prevalent in DMEK (*p* = 0.11). It was more common in FED for both surgical approaches. Only in BK treated with DSAEK were host steeper mean astigmatism (*p* = 0.03) and donors with smaller graft pre-cut diameters (*p* = 0.02) less likely to be related to GD. Regarding donor’s cause of death, only cardiovascular accident could be related to GD in BK treated with DMEK (*p* = 0.04). **Conclusions:** Our study shows that the conventional match between pathology and corneal lenticule is not sufficient to prevent ARB. Donor’s cause of death can impair graft and host attachment. In particular, cardiovascular death may impair the efficiency of donors’ endothelial cells, inducing GD after DMEK in BK.

## 1. Introduction

In 2016, in the USA, almost 17,000 corneal transplants were performed to treat Fuchs endothelial dystrophy (FED), one of the most common endothelial dystrophies among elderly patients in this country [1]. It causes an accelerated loss of endothelial cells and alterations in the Descemet Membrane that lead to corneal decompensation. FED differentiates into early- and late-onset forms. The latter manifests usually between the fourth and the fifth decade of life and is more prevalent in females, with F/M ratios of 2.5:1 and 3.5:1, respectively. The early-onset form, instead, usually becomes clinically evident in the first decade of life and is without a sex-based prevalence. FED causes painless visual disturbances in early and mild cases. In advanced stages, patients suffer from corneal edema and report blurred vision in the morning, especially after waking up, induced by nightly eyelid closure, which reduces corneal fluid evaporation. In later stages, patients may experience a painful rupture of epithelial bullae, associated with epiphora, blurred vision, and anterior chamber inflammation [2].

The only definitive treatment is keratoplasty, and this may be performed as Penetrating Keratoplasty (PKP) or Endothelial Keratoplasty (EK), in particular Descemet Stripping Endothelial Keratoplasty (DSAEK) and Descemet Membrane Endothelial Keratoplasty (DMEK). PKP constitutes a full-thickness corneal transplant and was the principal technique to manage FED before the advent of EK. Some authors have reported successful post-operative optically transparent cornea, in most cases with a full visual recovery after 2 years, usually associated with astigmatism and refractive errors that require further surgical procedures or the use of contact lenses.

On the other hand, EK techniques focusing the surgical approach only on the endothelial layer and preserving the remaining corneal structure provide benefits over PKP, namely, faster visual recovery, less astigmatism, stronger wound integrity, and a lower risk of immune rejection [3]. To achieve these results, a longer surgical learning curve is required [3].

Bullous keratopathy (BK) is a complication of several surgical procedures, such as cataract surgery, glaucoma surgery, and retinopexy procedures, but it may also be triggered by non-surgical conditions like anterior chamber tumors, congenital syndromes such as microcornea or ICE syndromes, neovascular or acute glaucoma, and herpetic endothelitis [4]. In cataract surgery, in particular, it may arise as a consequence of endothelial cell loss induced by intra-operative maneuvers. To our knowledge, one’s corneal endothelial cell count reduces from 7500 cells/mm^2^ at birth to 2500–2700 cells/mm^2^ in adulthood. Approximately 700 cells/mm^2^ are required to maintain transparency, and a further reduction to 300–500 leads to “critically low cell density”, resulting in the development of BK. Anatomically, it is characterized by endothelial cell polymegathism and pleomorphism, the abnormal production of the Descemet Membrane, and subsequent stromal edema [5].

The management of BK may be clinical, based on topical hypertonic agents such as sodium chloride (5%), based on anti-inflammatory drugs, based on topical and/or systemic antiglaucoma medications and corticosteroids, and/or based on lubricants or therapeutic contact lenses [5]. Today, corneal endothelial transplantation has become the gold standard in the management of corneal endothelial dysfunctions, replacing penetrating keratoplasty [6,7]. Since graft detachment (GD) is one of the earliest post-operatory complications of DSAEK and DMEK, usually recovered by re-inflating an air bubble in the anterior chamber to reattach the graft to the recipient stroma, the authors of this study investigated the predictive factors related to graft dehiscence.

## 2. Materials and Methods

We conducted a retrospective analysis of 72 eyes treated with DSAEK or DMEK for BK or FED at the UOC Oftalmologia Universitaria at Ospedale Universitario Consorziale Policlinico Giovanni XXIII di Bari, Italy, between January 2016 and March 2020, with the cut off being before the COVID-19 pandemic spread to Italy to avoid any possible COVID-related interference (Table 1 and Table 2). This study was performed in compliance with the principles of the Declaration of Helsinki. Donors’ tissues were processed by Fondazione Banca degli Occhi del Veneto ONLUS; Zelarino (Venice), Italy. The population involved in the study is not representative of the typical population affected by FED or BK.

All the procedures were performed under topical or local anesthesia. Technically, in both surgical techniques, the same surgeon (GA) performed a 2.4 mm clear corneal incision, then stripped the Descemet Membrane and endothelium layer with a reverse hook in the matter of marked and sized circular template applied on corneal surface to precisely visualize the border of the stripping layer. Intraoperative myosis was controlled by an intra-cameral acetylcholine chloride 1% solution to safely confine the donor’s lenticule in the anterior chamber. In both techniques, corneal lenticules were marked with an “F” to assure the proper positioning, ensuring that endothelial layer was correctly facing the anterior chamber. In DMEK, the rolled lenticule was managed by no-touch tapping and bubble techniques in the matter of repeated focal and rapid taps on the cornea in order to unfold the injected lenticules and to secure it to the recipient’s stroma by an air bubble injected in the anterior chamber. In both EK techniques, an inferior iridectomy was performed to prevent pupillary block. Post-operatively, patients were instructed to lie in a supine position for the following four hours. 

**Figure 1:**
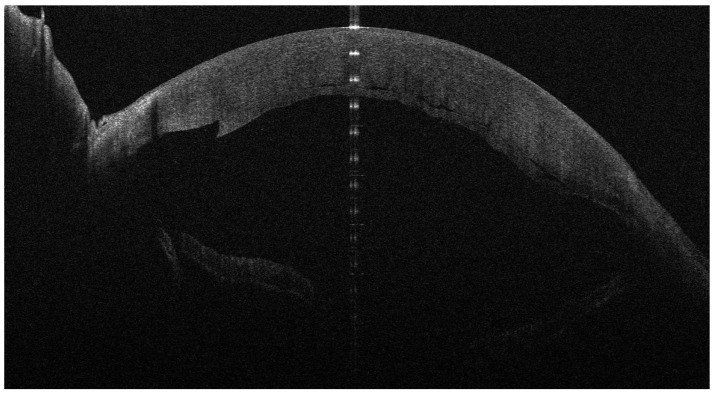
One day after DSAEK for FED; the implanted lenticule is mostly adherent and well positioned.

**Figure 2:**
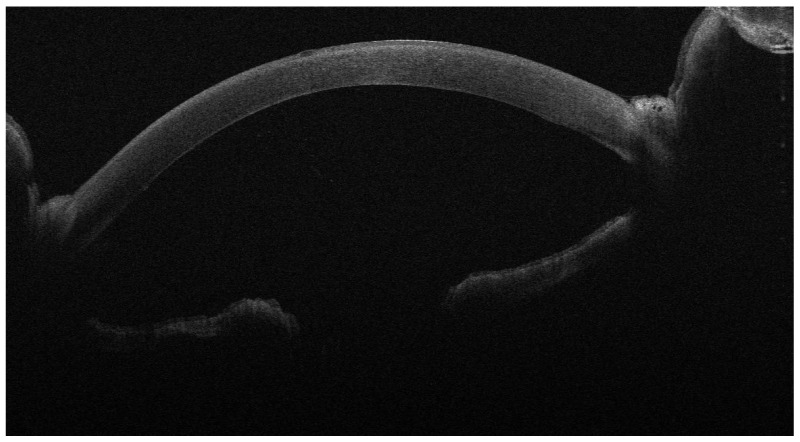
One day after DMEK for FED; the implanted lenticule is adherent and well positioned.

**Figure 3:**
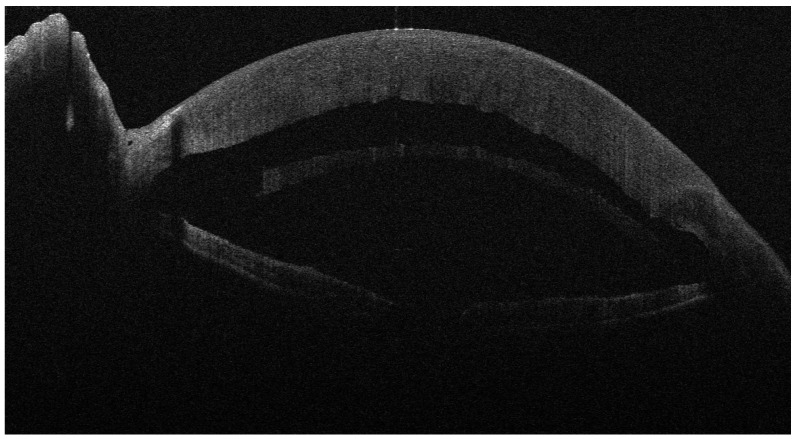
Four days after DSAEK for FED; the implanted lenticule is completely detached.

**Figure 4:**
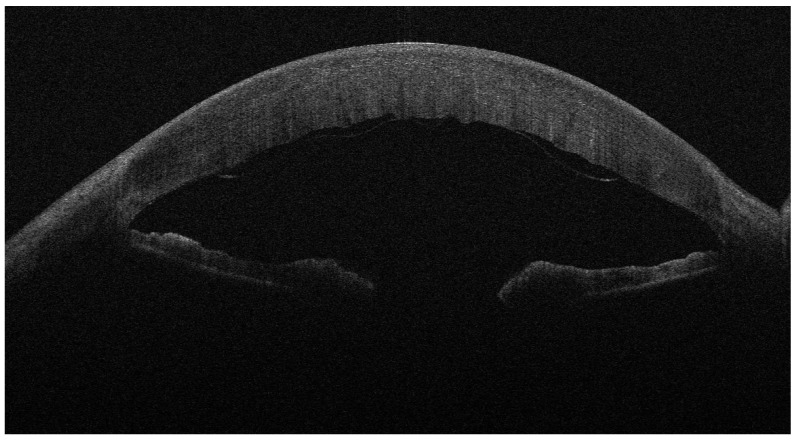
Four days after DMEK for FED; the implanted lenticule is detached.

At day 1 and 12 post operation, graft adherence to the host stromal bed was monitored by slit-lamp examination as well as by anterior segment optical coherence tomography (Anterior Segment OCT MS-39, Phoenix ver 4, CSO srl, Scandicci (Florence) Italy) (Figure 1 and Figure 2). If the graft was detached, defined by the presence of fluid between the recipient and donor cornea at any position of the cornea involving at least the 50% of the lenticule, it was immediately recovered under topical anesthesia by air bubble injection in the anterior chamber through the same clear corneal incision of the primary approach to relocate the donor lenticule to the stromal bed (Air Rebubbling—ARB) (Figure 3 and Figure 4). The main outcome was the occurrence of GD, treated with ARB. For each patient, we considered gender, age, period of corneal decompensation, pseudophakia, concomitant ocular pathologies, keratometry (steep axis and average keratometry), pachymetry, and combined lens phacoemulsification. Considering the data received with donors’ tissues, we also analyzed data about thickness, endothelial cell density, the diameter of the donor lenticule, the donor’s gender and age, their cause of death, and the time span between death and corneal explanation. Detailed features of patients and donors’ tissues are listed in in the tables. We adopted a vast and meticulous analysis of donors’ and receivers’ characteristics to investigate all the possible aspects that could influence GD, offering a detailed list of the examined parameters. Data were analyzed by a one-way ANOVA with Tukey’s post hoc test (*p* < 0.05) and a Chi-square test with an Odds Ratio calculation using GraphPad InStat3 (ver 3.06, GraphPad Software Inc. San Diego, CA, USA).

## 3. Results

**Figure 5:**
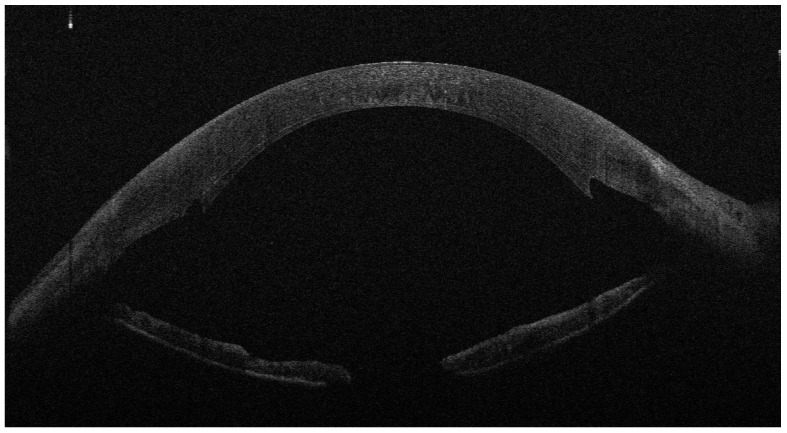
Eight days after ARB in DSAEK for FED; the implanted lenticule is attached.

**Figure 6:**
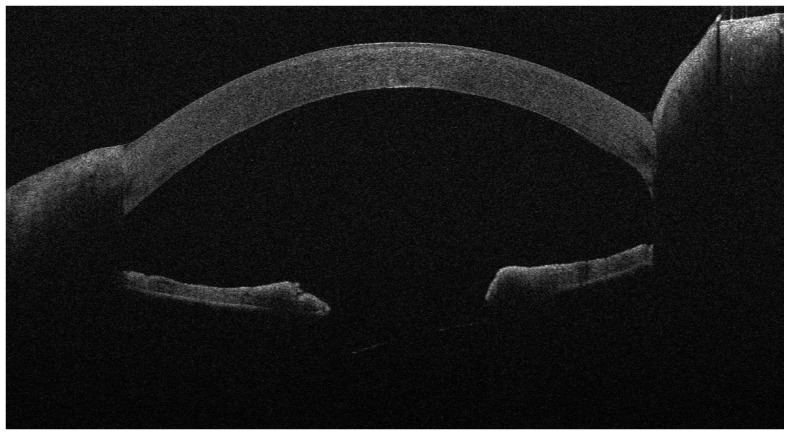
Ten days after ARB in DMEK for FED; the implanted lenticule is attached.

The patients were divided into two groups according to the surgical technique applied. The DSAEK group included 44 patients (M/F: 24/20, mean age: 66.66 ± 11.31 years), 44 eyes, while the DMEK group contained 28 patients (M/F: 9/19, mean age: 66.71 ± 9.66 years), 28 eyes. GD was most common but not significantly prevalent in DMEK, with 16 cases in 28 procedures (57.14%), higher than the prevalence in DSAEK, which was 11 cases in 44 procedures (25%) (*p* = 0.11, chi-square = 2.52, OR = 0.44, 95% CI: 0.18–1.08). It was more frequent in FED than in BK for both surgical strategies. GD was detected within 2 weeks from keratoplasty (Figure 2 and Figure 3) and was immediately treated with ARB, restoring the mechanical contact between the graft lenticule and host stromal bed (Table 3, Table 4, Table 5 and Table 6; Figure 5 and Figure 6). In the DSAEK group, ARB successfully recovered cases of GD that had occurred in 7 eyes affected by FED but only recovered cases of GD in 4 eyes affected by BK (*p* = 0.09, OR:0.28, CI 0.07–1.19); in the DMEK group, it successfully recovered cases of GD in 14 eyes overwhelmed by FED but only recovered cases of GD in 2 eyes affected by BK (*p* = 0.0008 OR:13, CI 2.58–65.57). Considering donors, we investigated the relations among the corneal lenticules characteristics and GD. In BK treated with DSAEK, host steeper mean astigmatism (43.86 ± 2.5 D, *p* = 0.03) and a smaller graft pre-cut diameter (9.6 ± 0.31 mm, *p* = 0.02) were less likely to be related to GD. The use of the DMEK approach for the same corneal disorder did not show any correlation with any of the investigated parameters (Table 3 and Table 4). In FED, none of the parameters were related to GD (Table 3 and Table 4). Cataract extraction combined with DSAEK or DMEK did not increase the risk of GD for both corneal disorders. Considering donor’s cause of death, only cardiovascular accident could be related to GD in BK treated with DMEK (*p* = 0.04, Chi-squared = 4.14, OD = 0.13, CI 0.0002–0.87) (Table 7 and Table 8).

## 4. Discussion

From the first successful human corneal transplant performed by Eduard Zirm (1887–1948) in 1905, corneal transplantation techniques have changed [8]. The years since and particularly the last 15 years have witnessed a significant increase in the demand for corneal transplants and grafts, especially for endothelial surgery [9]. Conversely, we face a significant shortage of donor corneas worldwide, to the point that, nowadays, only one cornea is available for potentially every seventy required [10]. As an early complication of Endothelial Keratoplasty, GD could be managed by ARB, but this involves costs and risks due to manipulation and endothelial cell loss.

Considering DMEK and DSAEK specifically to treat FED and BK some authors did not find any difference between the two techniques in terms of complications such as ARB [11], while others have found that donors’ medical histories (particularly regarding neoplasia [7], diabetes mellitus [12,13], age [14,15], endothelial cell density, and storage time of donor’s lenticules [9,16,17]) may affect surgical outcomes. Recently, Romano et Al. demonstrated that decreases in the adhesion forces and elastic modulii of the tissues of eye-bank pre-cut DMEK lenticules may contribute to increased GD rates [18]. In our study, anyway, we could not consider this finding because in EK we always implant eye-bank pre-cut tissues, limiting any counterpart experience of intra-operative donors’ lenticule preparation. All eyes were tamponed with a 100% air bubble injected in the anterior chamber both during EK and ARB. We did not consider any SF_6_–air mixture to reduce the bias of the study, despite the fact that some authors have found that the use of an air–gas mixture at various concentrations may lower the GD rate [19]. To our knowledge, in the literature, there is not any evidence of the effect of donor cause of death on GD after the deployment of corneal posterior lamellar techniques. In our study, 37.5% of all posterior lamellar keratoplasty patients developed GD and were scheduled for ARB (25% in DSAEK and 57.14% in DMEK, diverging from the data reported in [11,13,20,21]). Our results align with those of Marques RE et al., who found that GD was 2.5 times more common in DMEK than DSAEK in FED [22]. Our results found the same ratio in those with BK who received corneal lenticules from donors who died from cardiovascular failure. We hypothesized that the metabolic shock that corneal endothelial cells suffer in the hypoxic state following the vascular failure, associated with the mechanical trauma induced by the endothelial stripping procedure, could be responsible for endothelial impairment and consequent GD. Demsey et al. [23] demonstrated that graft dislocation is not influenced by variation in donor tissue processing and storage times. This evidence was extended to pre-cut tissue thanks to Dapena et al. [24]. A histopathological study of detached and failed grafts in DSAEK, conducted by Alkatan et al., reported a higher risk of GD in cases of irregular or thick grafts, graft–host interface fibrous/epithelial ingrowth, and interface infection [25]. Due to the retrospective nature of our study, we restricted our analysis to the thickness of DSAEK lenticules, finding that both BK and FED groups received similar grafts, avoiding any interference related to tissue preparation. Since ARB was successful and recovered all GD cases, we could not perform any further post-operative analysis. Some authors have considered corneal venting incisions to improve the adherence of a donor’s lenticule [26]; this strategy might increase the risk of deep infectious keratitis [27] or might induce irregular corneal astigmatism [28]. Moreover, Mohebbi assumed that venting incisions may not be necessary in uncomplicated DSAEK procedures [29]. Considering that we discharge patients the day after surgery, we did not perform any venting incisions to prevent any post-operative infections. Anterior segment biomicroscopy of all treated eyes did not reveal any sign of graft failure or graft rejection prior to or after ARB [30,31].

## 5. Conclusions

Despite the retrospective nature of this study, we focused our interest on donors’ characteristics. Our study shows that the conventional match between pathology and corneal lenticule is not sufficient to prevent ARB. Many hypotheses were discussed, but, most of the time, we were limited to corneal grafts. We extended the thread to donor cause of death because this factor can impair graft and host attachment. In particular, cardiovascular death may impair the efficiency of donors’ endothelial cells, inducing GD after DMEK in BK; this may be fully recovered by ARB performed within two weeks after posterior lamellar keratoplasty. We hope that our approach will inspire further investigations to refine and to identify the perfect match between donor and host in the prevention of ARB. We decided to limit our study to a period between January 2016 and March 2020, with the cut off being before the COVID-19 pandemic spread to Italy to avoid any possible COVID-related interference, offering “ante-COVID” data. Further investigations may use these data to assess the impact of COVID on early graft detachment in DSAEK and DMEK. 

## Figures and Tables

**Table 1:** Number of patients treated with rebubbling divided by pathology.

	FED	BK	ALL
Regular	20	25	45
Rebubbling	21	6	27
Total	41	31	72

**Table 2:** Number of patients divided by pathology and type of surgery.

	DSEK		DMEK	
	FED	BK	FED	BK
Regular	11	22	9	3
Rebubbling	6	4	14	2
Total	17	26	13	5

**Table 3:** Features of patients affected by BK, according to GD, in DSAEK and DMEK groups.

	DSAEK			DMEK		
	GD	No GD	*p*	GD	No GD	*p*
Age (years)	75.25 ± 14.53	64.73 ± 11.21	0.23	66.79 ± 9.63	67.44 ± 10.64	0.88
Kmean (D)	40.39 ± 3.79	43.86 ± 2.5	0.03	43.20 ± 1.39	44.91 ± 2.18	1
Kmax (D)	45.09 ± 2.97	45.6 ± 2.75	0.7	43.62 ± 1.03	46.3 ± 3.36	0.3
W-W (mm)	11.87 ± 0.36	11.69 ± 0.39	0.4	11.45 ± 0.15	11.65 ± 0.22	0.3
Time frame to GD (days)	8 ± 10.56			10.50 ± 13.44		

**Table 4:** Features of donors’ tissues, according to GD, for DSAEK and DMEK groups in BK.

	DSAEK			DMEK		
	GD	No GD	*p*	GD	No GD	*p*
Thickness (µm)	101.75 ± 22.17	110.41 ± 48.03	0.7	NA	NA	NA
Cell density (cells/mm^2^)	2625 ± 129	2609 ± 97	0.8	2600 ± 0	2566.67 ± 115. 47	NA
Pre-cut diameter (mm)	10.58 ± 0.74	9.6 ± 0.31	0.02	8 ± 0	8.00 ± 0.43	NA
Implanted lenticule diameter (mm)	7.94 ± 0.27	8.12 ± 0.31	0.3	8 ± 0	8 ± 0	NA
Donor age (years)	70.25 ± 6.5	66.73 ± 8.33	0.4	70.00 ± 2.83	75.33 ± 2.08	0.09
Combined cataract surgery,	2 (50%)	9 (22.70%)	0.42	0 (0%)	1 (33%)	NA
Time frame to tissue explant (hours)	9 ± 7.64	9.57 ± 6.62	0.9	9.00 ± 1.41	7 ± 3	0.5

**Table 5:** Features of patients affected by FED, according to GD, in DSAEK and DMEK groups.

	DSAEK			DMEK		
	GD	No GD	*p*	GD	No GD	*p*
Age (years)	68.14 ± 8.8	66.45 ± 10.78	0.7	66.79 ± 9.63	67.44 ± 10.64	0.88
Kmean (D)	44.54 ± 1.37	43.77 ± 1.70	0.3	43.74 ± 1.34	43.71 ± 1.45	0.96
Kmax (D)	45.34 ± 1.42	44.51 ± 1.60	0.28	44.34 ± 1.34	44.85 ± 1.82	0.45
W-W (mm)	11.37 ± 0.43	11.63 ± 0.59	0.33	11.72 ± 0.34	11.96 ± 0.25	0.09
Time frame to GD (days)	2.71 ± 2.14			6.86 ± 4.37		

**Table 6:** Features of donors’ tissues, according to GD, for DSAEK and DMEK groups in FED.

	DSAEK			DMEK		
	GD	No GD	*p*	GD	No GD	*p*
Thickness (µm)	104.43 ± 21.58	111.45 ± 17.22	0.45	NA	NA	
Cell density (cells/mm^2^)	2571.43 ± 111.27	2581.82 ± 98.16	0.84	2571.43 ± 82.54	2566.67 ± 70.71	0.89
Pre-cut diameter (mm)	9.36 ± 0.73	9.74 ± 0.92	0.38	7.96 ± 0.13	7.94 ± 0.17	0.75
Implanted lenticule diameter (mm)	8.0 ± 0.1	7.95 ± 0.19	0.6	8 ± 0	8 ± 0	NA
Donor age (years)	65.43 ± 8.79	64.27 ± 9.70	0.8	67.29 ± 5.24	70.44 ± 5.83	0.19
Combined cataract surgery,	6 (85.70%)	10 (90.90%)	0.76	13 [7.14%]	9 [100%]	1
Time frame to tissue explant (hours)	8.71 ± 7.16	13.55 ± 6.71	0.17	13.29 ± 6.57	10.33 ± 4.24	0.26

**Table 7:** Influence of donor cause of death on GD for DSAEK and DMEK in BK.

	BK—DSAEK		BK—DMEK		p		Odd Ratio	
Donor Cause of Death	GD	No GD	GD	No GD	p	Chi-Squared	Odds Ratio	95% CI
Neoplasm	2	11	0	1	0.67	0.18	0.65	0.02–21.2
Cardiovascular	0	7	2	0	0.04	4.14	0.13	0.0002–0.87
Cerebrovascular	1	1	0	1	0.37	0.75	3	0.06–151.34
Respiratory	1	3	0	0	0.58	0.31	1.29	0.03–53.56
Traumatic	0	0	0	0	NA			
Dysmetabolic	0	0	0	1	NA			
Unknown	0	0	0	0	NA			
Total	4	22	2	3	0.51	0.43	0.27	0.03–2.19

**Table 8:** Influence of donor cause of death on GD for DSAEK and DMEK in FED.

	FED—DSAEK		FED—DMEK		*p*		Odd Ratio	
Donor Cause of Death	GD	No GD	GD	No GD	*p*	Chi-Squared	Odds Ratio	CI
Neoplasm	4	4	10	3	0.43	0.63	0.3	0.04–2
Cardiovascular	3	4	4	3	0.59	0.29	0.56	0.07–4.68
Cerebrovascular	0	1	0	0	NA			
Respiratory	0	0	0	0	NA			
Traumatic	0	2	0	2	NA			
Dysmetabolic	0	0	0	1	NA			
Unknown	0	0	0	0	NA			
Total	7	11	14	9	0.28	1.17	0.41	0.11–1.45

## Data Availability

Data are stored at Main Office of Azienda Ospedaliera Universitaria Consorziale Policlinico Giovanni XXIII di Bari, 70100, bari, Italy.
